# Potential antibacterial activity of some Saudi Arabia honey

**DOI:** 10.14202/vetworld.2017.233-237

**Published:** 2017-02-21

**Authors:** Ahmed G. Hegazi, Faiz M. Al Guthami, Ahmed F. M. Al Gethami, Fyrouz M. Abd Allah, Ashraf A. Saleh, Ehab A. Fouad

**Affiliations:** 1 Department of Zoonotic Diseases, National Research Centre, Dokki, Giza, Egypt; 2 Al Guthami foundation, Saudi Arabia; 3 Department of Parasitology, National Research Centre, Dokki, Giza, Egypt; 4 Department of Microbiology and Immunology, National Research Centre, Dokki, Giza, Egypt

**Keywords:** antibiotic-resistant, potential antibacterial activity, Saudi Arabia honey

## Abstract

**Aim:**

The aim of this study was to investigate the potential antibacterial activity of some Saudi Arabia honey against selected bacterial strains of medical importance.

**Materials and Methods:**

A total of 10 Saudi Arabia honey used to evaluate their antimicrobial activity against some antibiotic-resistant pathogenic bacterial strains. The bacterial strains were *Staphylococcus aureus*, *Streptococcus pyogenes*, *Klebsiella pneumoniae*, *Escherichia coli*, and *Pseudomonas aeruginosa*.

**Results:**

The antibacterial activity of Saudi honey against five bacterial strains showed different levels of inhibition according to the type of honey. The overall results showed that the potential activity was differing according to the pathogen and honey type.

**Conclusion:**

It could be concluded that the Saudi honey inhibit the growth of bacterial strains and that honey can be used as complementary antimicrobial agent against selected pathogenic bacteria.

## Introduction

The microbial resistance to antibiotics and chemicals has been increased worldwide against harmful microorganisms [1,2].

Honey has been used as the oldest sweeter since ancient times as a nutritive as well as effective remedy [3,4], antibacterial [5-8], also honey is recognized as an effective antimicrobial agent used topically in the treatment of burns and wounds [9-12], dyspepsia, peptic ulcer [13,14] and gastritis [15-17], and liver disease [18].

The biological properties of honey play an important role due to its floral source [19]. There are several factors attributed to antimicrobial activity of honey [17,20,21] as endogenous hydrogen peroxide content [11,22], inhibin [23] which acts as antibacterial factor other than H_2_O_2_ [24], hydrogen peroxide [25], osmotic effect of honey, the low pH [20,26], defensin-1, as well as the presence of phytochemical factors [27,28], phytochemical components [17,24,29,30]. Some of the phytochemical components of honey could stimulate monocytes to release cytokines as interleukin (IL)-1 and IL-6, tumor necrosis factor-alpha, which modulate the immune response to overcome the infection [4,31,32]. The antibacterial activity of different honey was studied by many several authors [6,7,8,20,21,33-36], many honey are available in the Saudi market either locally produced by Saudi beekeepers or imported from different countries [8,35,36]. A comparison between Saudi Arabia honey and Egyptian honey was previously studied by Hegazi [7].

Thus, this investigation was evaluated the potential antibacterial activity of 10 Saudi Arabia honey against some bacterial strains of medical importance.

## Materials and Methods

### Ethical approval

Experiments were performed according to the Guide for the care and use of Laboratory animals and Ethical Approval of Animal Rights according to Committee, National Research Centre, Egypt.

### Bacterial strains

Five pathogenic bacterial strains have antibiotic-resistant. Gram-positive and Gram-negative were used. The Gram-positive bacteria were including *Staphylococcus aureus* (ATCC 25923) and *Streptococcus mutans*. The *S. mutans* strain was provided with Cairo Microbiological Resources Center (Cairo MIRCEN). The Egypt Microbial Culture Collection number for the *S. mutans* is 1815^T^ where the Gram-negative bacteria included *Klebsiella pneumoniae* (ATCC 27736), *Escherichia coli* (ATCC 35218) and *Pseudomonas aeruginosa* (ATCC 27853).

### Honey

Fresh 10 Saudi honey samples (1 kg each) were kindly provided by Alnahal Aljwal Company, 2015 flowering season). The monofloral honey harvested from apiaries (From Authorized proved apiary farm of Alnahal Aljwal, Saudi Arabia), these honey are vended as “monofloral” meaning that the honey must derive from at least 55% of pollen from a single floral source according to Louveaux *et al*. [37]. The collected honey samples were Shafallah honey (*Capparis spinosa*), acacia (*Acacia nilotica*) honey, Astragalus honey (*Astragalus pelecinus*), Talh honey (*Thymus vulgaris*), Sidr honey (*Ziziphus spina-christi*), spring Lena honey (*Rhanterium epapposum*), large influx honey (*Acacia tortilis*), olive (Alaatm) honey (*Olea europaea*), Dahbianh honey (*Carduus acicularis*), and Citrus honey (*Citrus sinensis*). Each honey sample was collected in a sterile universal glass container and kept at 2-8°C until tested. Physiological saline PBS pH 7.2 was used for all dilution steps under aseptic condition according to the method described by Nzeako and Hamdi [38]. Evaluations of the antibacterial activity of different honey dilution were performed according to Hegazi and Allah [7,39]. The results of antibacterial activity against different examined bacteria were determined.

### Antibacterial assays

Five bacterial strains were used: *S. aureus* (ATCC 25923), *S. mutans* (1815^T^), *K. pneumoniae* (ATCC 27736), *E. coli* (ATCC 35218), and *P. aeruginosa* (ATCC 27853). The bacterial suspension was adjusted by comparison of 0.5 Mc-Farland turbidity standards (5 × 10^7^ cells/ml). Then, it was further diluted to obtain a final of 5 × 10^6^ cells/ml. These bacterial strains were enriched on selective broth for bacterial propagation [40]. In a separate tube containing 40 µl of 21.30% honey [34] concentration mixed with 0.20 µl/10 ml from inarched broth of each propagated *S. aureus, S. mutans*, *K. pneumoniae, E. coli*, and *P. aeruginosa*. These tubes were incubated at 37°C for 24 h. The growths of control bacterial strains as well as inhibitions of the bacterial growth due to mixed with honey were measured by turbidity at 420 nm wavelength. The mean values of inhibition were calculated from triple reading in each test [7].

### Statistical analysis

Data were analyzed statistically using student “T” test showing mean + standard deviation. Data were compared using one-way. Statistical significance was accepted at p<0.01 according to Zar [41].

## Results

**Figure-1:**
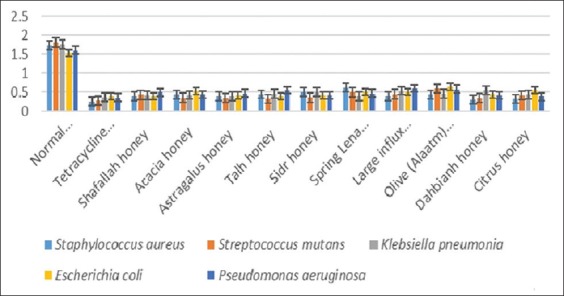
Results of efficacy of different honey types against pathogenic bacteria.

The results of the different Saudi honey induced growth inhibition of *S. aureus, S. mutans*, *K. pneumoniae, E. coli*, and *P. aeruginosa* were illustrated Table-1 and Figure-1. All honey types at concentration of 20.30% showed inhibition of different bacterial growth. The efficiency of Tetracycline (50 µg) was indicated that the inhibition of *S. aureus* (0.253±0.001) and *S. mutans* (0.371±0.001), *K. pneumonia* (0.362±0.001), *E. coli* (0.396±0.002), and *P. aeruginosa* (0.351±0.001).

**Table-1:** Results of efficacy of different honeys types against pathogenic bacteria.

Bacteria antibacterial agent	*S. aureus*	*S. mutans*	*K. pneumonia*	*E. coli*	*P. aeruginosa*
Normal bacterial growth	1.721±0.001	1.807±0.002	1.746±0.001	1.528±0.011	1.601±0.002
Tetracycline (50 µg)	0.253±0.001	0.271±0.001	0.362±0.001	0.396±0.002	0.351±0.001
Shafallah honey	0.384±0.015	0.424±0.001	0.404±0.002	0.398±0.001	0.494±0.001
Acacia honey	0.440±0.001	0.353±0.011	0.421±0.031	0.532±0.012	0.435±0.012
Astragalus honey	0.394±0.002	0.345±0.014	0.383±0.001	0.402±0.001	0.467±0.001
Talh honey	0.445±0.002	0.317±0.001	0.461±0.014	0.399±0.002	0.549±0.002
Sidr honey	0.497±0.003	0.359±0.001	0.501±0.001	0.411±0.011	0.411±0.011
Spring Lena honey	0.621±0.001	0.491±0.001	0.381±0.031	0.499±0.001	0.462±0.012
Large influx honey	0.394±0.002	0.444±0.002	0.533±0.001	0.501±0.002	0.595±0.001
Olive (Alaatm) honey	0.425±0.002	0.595±0.002	0.451±0.014	0.634±0.014	0.579±0.002
Dahbianh honey	0.299±0.003	0.347±0.003	0.551±0.001	0.432±0.012	0.411±0.011
Citrus honey	0.319±0.013	0.414±0.011	0.439±0.016	0.558±0.001	0.382±0.003

*S. aureus*=*Staphylococcus aureus*, *E. coli*=*Escherichia coli*, *P. aeruginosa*=*Pseudomonas aeruginosa*, *K. pneumonia*=*Klebsiella pneumonia*

The honey inhibition of *S. aureus* ranged from 0.299±0.003 to 0.621±0.001 and *S. mutans* ranged from 0.317±0.001 to 0.595±0.002. The highest inhibition of *S. aureus* against Dahbianh honey was 0.299±0.003 and the highest inhibition of *S. mutans* against Talh honey was 0.371±0.001, where the lowest inhibition of *S. aureus* against Spring Lena honey was 0.299±0.003 and the lowest inhibition of *S. mutans* against olive (Alaatm) honey was 0.595±0.002.

The highest antibacterial activity was determined in Spring Lena honey (0.381±0.031) against *K. pneumonia*, while Shafallah honey (0.398±0.001) against *E. coli* but Citrus honey (0.382±0.003) against *P. aeruginosa*, where the lowest activity was observed in Dahbianh honey (0.551±0.001) against *K. pneumonia*, while Olive (Alaatm) honey (0.634±0.014) against *E. coli* but large influx honey (0.595±0.001). It was clear that all honey types induced an inhibitory activity of the growth of different pathogens. This reduction depends on the type of honey.

## Discussion

The investigation into antibacterial activity of Saudi honey from different sources of 5 pathogenic bacteria was recorded in Table-1 and Figure-1. 20.30% honey concentration from different types showed inhibition of five bacterial growths. The inhibition of these bacteria may depend on the type of honey origin. These results were attributed to the floral source of honey which acts an important role on its biological properties [19]. The antimicrobial activity of honey also return to several factors [17,20,21] as osmotic effect of honey [20,26,42]. Acidity of honey (pH range from 3.2 to 4.5) or activity of glucose oxidase in the ripening of nectar [43]. The presence of hydrogen peroxide [44,45], endogenous hydrogen peroxide content [11,22], inhibin [23] which acts as antibacterial factor other than H_2_O_2_ [24], hydrogen peroxide [25], non-peroxide substances [46,47], defensin-1, as well as the presence of phytochemical factors [27,28] and phytochemical components [17,24,29,30]. The antibacterial activity of different honey was studied by several authors [5-8,20,21,33-36,48].

Comparison between Manuka honey with ling heather honey was determined by Lu Hodgeson [49] who found that whereas *S. aureus* and *P. aeruginosa* were inhibited by both honey. While, ling heather honey was inhibited *E. coli, Proteus mirabilis* and *Streptococcus faecalis*, on the other hand, yet Manuka honey was inhibited *E. coli, P. mirabilis* and *S. faecalis*. Media containing various concentrations of honey was evaluated against Gram-positive and Gram-negative bacteria [16] and they found that most pathogenic bacteria failed to grow in honey at a concentration of 40% or above. Hegazi and Allah [8] found that honey samples with different Saudi honey, were effective antibacterial against different examined pathogenic bacteria. Several honey available in the Saudi market especially the locally produced Shaoka, and Taify Sidr, in addition to imported Yemeni Sidr, black seed, clover and orange blossom are as potent as Manuka honey [36]. Furthermore, 10 honey samples collected from different floral areas around Riyadh were investigated [3]. 9 widely used honey in Saudi Arabia (Yemeni Sidr, Taify Sidr, Kashmiri Sidr, Shaoka, Somra, Black Seed, Black Forest, and Clover honey), and Manuka honey against *E. coli*, *P. aeruginosa*, *Salmonella enterica* serovar Typhimurium, *Shigella flexneri* and *K. pneumoniae*, *S. aureus*, and *Streptococcus pyogenes* were examined by Halawani and Shohayeb [35]. The most sensitive Gram-negative bacterium was *P. aeruginosa* while the most sensitive Gram-positive bacterium was and *S. pyogenes* [36]. Honey from some countries as Manuka honey from Australia, heather honey from the United Kingdom, and locally marketed Indian honey was detected their antibacterial activity [50]. Honey obtained from Izmir proved more effective as inhibitors against *P. aeruginosa, E. coli* and *S. aureus*, where the honey obtained from Muğla exhibited high anticandidal activity on *C. albicans* [22].

Finally, we could have concluded that the variations in the activity of different honey were attributed to the previously mentioned factors which influenced the antibacterial activity [7] as osmotic properties of honey [20,39]; honey pH or activity of glucose oxidase [41]; hydrogen peroxide [42,45], non-peroxide substances [46,47], presence of propolis which contain flavonoid [46], and volatile antibacterial substances [40].

## Conclusion

From the current results, it concluded that the Saudi honeys inhibit the growth of bacterial strains and that honey can be used as complementary antimicrobial agent against selected pathogenic bacteria.

## Authors’ Contributions

AGH, give the idea, share as well as supervise on practical work, editing and help publishing of the article. FMA and AFMA, provided some materials and help in editing the article. FMAA, AAS and EF. They make isolation and identification and do the practical work, help during editing and publishing of the article. All authors have read and approved the final manuscript.
